# Book Review

**Published:** 1935

**Authors:** 


### Reviews of Books

**Constructive Eugenics.** By M. Siegel, M.D. Pp. xii., 196. Toronto: McLelland and Stewart Ltd. 1934. Price $2.50.—Those unversed in the elements of eugenics, in fact any intelligent boy or girl from sixteen upwards, will find this book a good introduction to further reading on this subject. It gives a sufficient, if superficial, outline of heredity and the Nature versus Nurture controversy. It concerns itself with the elucidation of the misunderstandings and wrong interpretations of much which has been written on eugenics. It discusses, analyses and suggests solutions for the more important problems of this subject of which all should be aware. The second half of the book is devoted to a constructive eugenic programme, pointing out errors that have arisen in such matters as segregation and sterilization, their unwise application, and the means of avoiding like errors in the future. It is up to date and on the whole sound, and though it formulates little that is new, gives in a clear and constructive manner the author's conclusions on a subject which has long been the source of much discussion, and in the incomplete state of our present knowledge is likely to remain so for many years to come.

**Clinical Methods.** Tenth Edition. By R. Hutchison, M.D., LL.D., and D. Hunter, M.D. Pp. xiii., 658. Illustrated. London: Cassell & Co. Ltd. 1935. Price 12s. 6d.—This is the tenth edition of a book which has been the standard guide and counsellor of clinical clerks for the past thirty years. The fact that new editions and reprintings follow each other so rapidly is sufficient proof, if any be needed, of its popularity. In this edition the whole has been revised and special attention has been paid to the chapters on the urine, the blood and the nervous system. Enlargement of the book has been avoided by rigorously discarding all out of date matter. The result is a small, handy volume which no clinical clerk should be without, and which will continue to be of the greatest help and value for long after he is qualified.

**Religion and Psychotherapy.** By A. G. IKIN, M.Sc. Pp. 139. London : Student Christian Union Press. 1935. Price 3s. 6d.—The object of this little book is to plead for closer co-operation between doctors, clergy and psychologists in the work of psychotherapy. The author, who is described as "an expert practising psychologist," is not himself a member of the medical profession, but is evidently accustomed to working in the closest collaboration with both doctors and clergy. He makes the obvious point that neuroses and psychoses are affecting an ever increasing number of the population, that all of these disorders are the result of maladjustment to environment and to the circumstances of life. Amongst these must be placed a large number of cases where the maladjustment is associated with the realm of religion and with conflict in things of the spirit. The writer would approach these problems partly along the religious and partly along the psychological plane. He makes an eloquent plea for the admission to the Diploma of Psychological Medicine of non-medical students of psychology, including ordinands. He considers that certain clergy who would command the respect and support of the medical profession should be definitely set apart for the work of psychotherapy, whilst at the same time he is sympathetically alive to the natural distrust of our profession for charlatans and untrained enthusiasts. In his own work he would appear to practise a sort of blend of the principles of Freud, Jung and Adler. There is an interesting and provocative chapter on Spiritual Healing, but it is not quite clear (at any rate to the reviewer) whether he would ascribe all the effects to suggestion, plus activities of the hormones, or whether there is something added to any factors in the healer and in the healed. There are many references to psychological works and to the Bible. Are all of these quite accurate ? Was it Christ who said : "As a man thinketh in his heart, so is he" ? Was it not one of the proverbs of Solomon ? An interesting, provocative — but nowhere provoking — book.

**Norwood Sanatorium—Report for 1932, 1933 and 1934.** By H. K. V. SOLTAU, M.D. Pp. 13. Illustrated.—The records of Beckenham and Rendlesham now cover 7,658 cases, and are of the greatest value and interest to all concerned with the study and treatment of alcoholism. The increasing number of young persons treated is, in the opinion of the medical superintendent, due to the lack of parental control during the war period. The causative factors in alcoholism are given in the following order: Heredity, domestic worry and friction, insomnia, financial worries, business anxieties, and in women menstrual troubles. Amongst the drug addicts an increasing number are victims to the barbiturates. Dr. Soltau is not in favour of the sudden withdrawal of alcohol, especially in patients with a high tolerance. The drugs employed in treatment are atropine with strychnine, and apomorphine. Research is being carried out in connection with the absolute percentage of ethylic alcohol in the blood in relation to the amount drunk, and the significance of low blood sugar content in association with the desire for alcohol and the possibilities of treatment of this symptom by insulin and glucose.

**Radium and Cancer.** By H. S. Souttar, D.M., M.Ch. Pp. xiii., 387. Illustrated. London: William Heinemann Ltd. 1934. Price 21s.—This is one of the clearest and best books on radium therapy which has been published in English. The lay-out of the book is excellent. The reader is immediately reminded of the pathology, etc., of each lesion, and the wide margin leaves ample room for any additional note one would like to make. The diagrams, although not numerous, are admirable, and while no extravagant claims have been made for radium, the author throughout has tried to assess its proper value in the treatment of malignant disease. The chapter on physics is clear, but might be further improved by fuller information about dosage, including isodose curves and the international "*r*" unit. If there is any fault to find in this book it is perhaps that Mr. Souttar is a little dogmatic and does not give sufficient authority for some of his remarks, as, for example, on page 306, where he mentions that no organism can grow in close proximity to radium emanation. But on the whole one has nothing but praise for this book, which ought to be in the hands of all who practise radium therapy.

**The Conduct and Fate of the Peripheral Segment of a Divided Nerve When United to Another Divided Nerve.** By Sir Charles Ballance, K.C.M.G., C.B., M.V.O. Pp. 45. Illustrated. London: Macmillan and Co. Ltd. 1934. Price 7s. 6d.—This short report should be read by all who are interested in the question of nerve regeneration and of the nerve-supply of muscles. In a previous communication the author has shown that a sensory nerve can apparently take over all the functions of a motor one. Here he demonstrates that the cervical sympathetic fibres can grow down the degenerated axons of a divided somatic motor nerve, produce regeneration of the motor end plate and muscle fibres, and restore apparently normal function !

**Standard Classified Nomenclature of Disease.** Second Edition. By H. B. LOGIE, M.D., C.M. Pp. xxi., 870. New York: The Commonwealth Fund. 1935. Price 15s. —The appearance of a second edition of this work testifies to the popularity which it has enjoyed in the land of its origin. The system is particularly adapted to those organizations that use a punch-card system for the tabulation of diseases ; but it must be disconcerting for those such users to learn that whole sections have been entirely rewritten. However, the arrangement is ingenious and thorough, and seems to appeal to a large number of users in America.

**Gould's Medical Dictionary.** Fourth Edition. Edited by R. J. E. SCOTT, M.D., and C. V. BROWNLOW. Pp. xviii., 1,538. Illustrated. London: H. K. Lewis & Co. Ltd. 1935. Price 30s.—The appearance of yet another edition of this standard work testifies to its continued popularity, and affords some support to the author's claim that " in post-graduate life and practice there is no book that is so frequently consulted." By careful editing space has been made for the inclusion of new words without increase in size : in addition there are 174 tables of acids, arteries, bacilli, bones, operations, etc. For every word there is given a definition and pronunciation, and for each drug the dose and indications. What do the advocates of " Nudism " think of the definition, " A morbid tendency to remove clothing observed in mental disease " ? The old difficulties of spelling still divorce " enomania " and " oenilism," and " ethocain " seems to have been lost in transit ; while on p. 328 " albuminum " for " aluminum " reads curiously. But the rarity of misprints and errors of any kind is evidence of the great care expended in the preparation of this dictionary, and its excellent arrangement, type and paper should retain it in the unique position it has long occupied.

**The Treatment of Fractures.** Fourth Edition. By Dr. LORENZ BÖHLER, M.D. English translation by E. W. HEY GROVES. Pp. 578, 1059 figures. Bristol: John Wright & Sons Ltd. 1935. Price 42s.—The name of Lorenz Böhler is a household word among those who treat fractures of all types. This work has been published at a time when great interest has been aroused in the subject by the Fracture Committee of the British Medical Association, and at last it is being realized that the treatment of fractures is a highly specialized branch of surgery. The translator has been careful to keep to the continental type of text-book, where every detail is described with great accuracy. After reading it one is impressed with the way in which the author pays attention to the minutest detail. Each fracture is taken separately and described fully and the possible causes of failure enumerated. But the great merit of Böhler's pioneer work consists neither in attention to detail nor in originality of method, but in his recognition of traumatic surgery and especially that of fractures as a special branch of surgery. In his "Unfall Krankenhaus" he has a hospital of 120 beds with a large out-patient department devoted solely to the treatment of accident cases and financed entirely by the National Insurance Society. Under this plan the cost of compensation to the Society is only one-third of what it was when accident cases were treated in the former haphazard fashion. Böhler in his team-organization and follow-up system has proved beyond all doubt that unity of control, continuity of treatment and a thorough knowledge of ultimate functional results are essential for this work. It would have been a great improvement if the subject-matter which has been confined to Appendix B and C had been left out. It is a pity also that the plaster technique has not been separately described. If the orthopædic chapter had been omitted (*i.e.* Appendix C) and the technique of the unpadded plaster had been included the book would have been much more valuable. There are many excellent illustrations with full descriptions. This is undoubtedly the best work that has yet been produced on the treatment of fractures.

